# Identification of AAV serotypes for gene therapy in Krabbe iPSCs-derived brain organoids

**DOI:** 10.1016/j.gendis.2024.101269

**Published:** 2024-03-19

**Authors:** Yafeng Lv, Zhi Cui, Hongbo Li, Jing Wang, Mulan Wei, Yuanlang Hu, Xun Li, Chunyu Cao, Ye Zhang, Wei Wang

**Affiliations:** a Hubei Key Laboratory of Tumor Microenvironment and Immunotherapy, College of Basic Medical Sciences, China Three Gorges University, Yichang, Hubei 443000, China; b State Key Laboratory of Medical Molecular Biology, Department of Biochemistry and Molecular Biology, Institute of Basic Medical Sciences, Chinese Academy of Medical Sciences & Peking Union Medical College, Beijing 100005, China; c Yiling People's Hospital of Yichang, Yichang, Hubei 443000, China; d China-Japan Friendship Hospital, Beijing 100029, China

Krabbe disease, also known as globoid cell leukodystrophy, is a rare lysosomal storage disorder. It is primarily caused by mutations in the *GALC* gene on chromosome 14q31, leading to GALC enzyme deficiency in lysosomes. This results in the accumulation of toxic substrate psychosine in the nervous systems.^1^ Currently, hematopoietic stem cell transplantation is the only available treatment, offering only a delay in neurological deterioration. Gene therapy, particularly using recombinant adeno-associated viruses (AAVs), shows promise for treating genetic diseases by introducing functional genes into target cells.^2^

Human brain organoids, particularly those derived from patient-specific induced pluripotent stem cells (iPSCs), offer a compelling model for recapitulating the physiological and pathological features of the human brain. We previously developed a model utilizing iPSCs (K-iPSCs, PUMCi001-A) from a patient with Krabbe disease.^3^ In the current study, we have advanced this work by generating brain organoids from the K-iPSCs. The induction process and critical stages are illustrated in Figure 1A. Following a differentiation period of six weeks, we identified the formation of an SOX2^+^ ventricular zone-like layer and a TUJ1^+^ neuronal layer within the brain organoids (Fig. 1B). Further histological analysis of the dorsal cortex region in the organoids, employing pax6^+^ radial glial cells and tuj1^+^ neuron markers, revealed a characteristic tissue architecture akin to the zone-like layer, with neurons positioned at the basal surface. Moreover, MAP2 immunostaining, indicative of neuronal presence, confirmed the existence of a basal neural layer that resembles the preplate. The detection of KI67, a cell proliferation marker, across numerous cells corroborated the active proliferation of radial glial cell-like cells in the zone-like layer. We also observed the distribution of astrocytes and oligodendrocytes in the Krabbe iPSC-derived brain organoids (Fig. 1B). Genomic sequencing of the brain organoids allowed us to identify mutation sites within the GALC gene, characteristic of Krabbe disease, in the organoid model (Fig. S1). The hallmark biochemical features of Krabbe disease, namely reduced GALC enzyme activity and elevated levels of the GALC substrate psychosine, were quantitatively assessed. The enzyme activity of GALC was markedly diminished, and psychosine content was significantly elevated in the Krabbe organoids compared with those derived from the H9 human embryonic stem cell line (Fig. S2, 3). These findings demonstrate that the brain organoids generated in this study not only mirror the structural attributes of brain tissue but also faithfully maintain the genetic and biochemical hallmarks of Krabbe disease, thus validating their application as a disease model.

**Figure 1:**
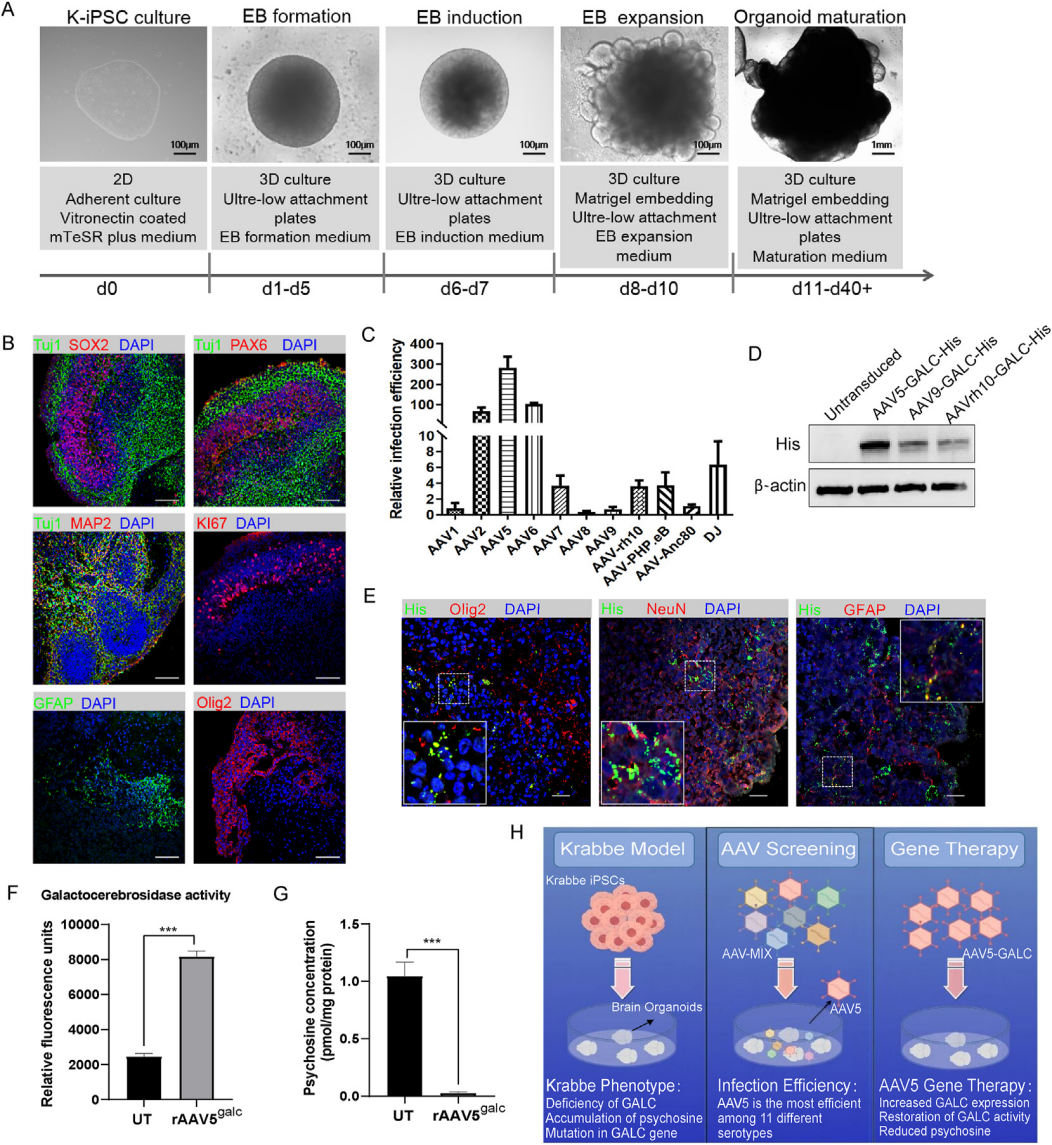
Establishment of Krabbe iPSCs-derived brain organoids and identification of AAV serotypes for gene therapy. **(A)** Timeline of the protocol for generating brain organoids and representative images at different stages. Scale bar: 100 μm. **(B)** Immunostaining for neural progenitor marker SOX2, neuronal markers MAP2 and Tuj1, ventricular zone marker PAX6, proliferation marker KI67, astrocyte marker GFAP, and oligodendrocyte marker Olig2 in Krabbe brain organoids at day 45 of culture. Nuclei were stained with DAPI. Scale bars: 100 μm. **(C)** Relative infection efficiency in Krabbe brain organoids on day 7 after AAV transduction. Organoids were microinjected with 1E9 GC of AAVmix genome copies (GC). After one week, RNA was extracted from the organoids, and quantitative PCR was then conducted to determine the proportion of AAV in the organoids. AAVmix viral genome served as a control. **(D**) Western blot analysis showed that transduction of AAV5-hGALC resulted in higher expression levels of GALC compared with untransduced brain organoids (UT), AAV9-hGALC, and AAVrh10-hGALC. **(E)** Immunostaining for Krabbe brain organoids with antibodies against His (tagged with GALC), NeuN, Olig2, and GFAP after AAV5-hGALC virus infection. **(F)** AAV5-hGALC virus rescued the GALC enzyme activity in Krabbe brain organoids. Data were expressed as mean ± standard deviation (*n* = 3). ∗∗∗*P* < 0.001. **(G)** AAV5-hGALC virus decreased the accumulation of the GALC substrate psychosine in Krabbe brain organoids. Data were expressed as mean ± standard deviation (*n* = 3). ∗∗∗*P* < 0.001. **(H)** Graphical summary. The figure was built using resources from Figdraw. iPSCs, induced pluripotent stem cells; AAV, adeno-associated virus.

AAVs are extensively utilized as vectors for gene therapy in monogenic disorders.^4^ However, the infectivity of different AAV serotypes in the human brain has not been comprehensively studied. In our investigation, we employed Krabbe patient-derived brain organoids to screen for AAV serotypes with high infection efficiency in human brain tissue. A mixture of AAV serotypes, including AAV1, AAV2, AAV5, AAV6, AAV7, AAV8, AAV9, AAVrh10, AAV-PHP.eB, AAV-Anc80, and AAV-DJ, each carrying a unique DNA barcode, was introduced into the organoids. Seven days post-infection, we extracted total RNA from the organoids and quantified the prevalence of each AAV serotype using quantitative PCR, with the viral genome of the AAV serotype mixture serving as a reference (Fig. 1C). Our analysis indicated that AAV5 was the most efficient in transducing the brain organoids, followed by AAV6 and AAV2. To ascertain the efficacy of the hGALC-His fusion protein as a therapeutic strategy in Krabbe disease models, we engineered AAV serotype 5 (AAV5) vectors harboring the construct for a 6xHis-tagged GALC (AAV5-CBA-hGALC-His). Following transduction of the Krabbe brain organoids, we observed a significant up-regulation in GALC expression in comparison to the untransduced controls, AAV9-CBA-hGALC-His and AAVrh10-CBA-hGALC-His, as depicted in Figure 1D. Immunofluorescence analysis further validated the intracellular expression of the hGALC-His protein within the organoids, and co-staining demonstrated that AAV2/5 can efficiently transduce neurons (stained with NeuN), oligodendrocytes (stained with Olig2), and astrocytes (stained with GFAP) within the organoids (Fig. 1E). The co-staining confirms that AAV2/5 has a broad tropism within the central nervous system cellular environment. Subsequent biochemical analyses were conducted to measure the GALC enzyme activity and psychosine levels, which serve as critical biomarkers for Krabbe disease. Treatment with AAV5-CBA-hGALC-His was shown to restore GALC enzyme activity and markedly reduce the psychosine accumulation (Fig. 1F, G), suggesting substantial mitigation of the biochemical pathology associated with Krabbe disease and underscoring the therapeutic potential of this gene therapy approach.

This investigation successfully generated a human brain organoid model from the iPSCs of a patient with Krabbe disease. The model adeptly replicated the key pathological hallmarks of the disease, both structurally and biochemically, thus providing an invaluable platform for in-depth mechanistic studies and therapeutic screenings. The brain organoid model offers several advantages over traditional animal models: it more accurately represents the human brain's architecture and cellular composition, which is crucial for studying a human-centric pathology like Krabbe disease; it retains the patient-specific genetic background, ensuring the preservation of the disease's genetic intricacies; and it circumvents the ethical and immunological complications often associated with animal research, thereby facilitating preliminary gene and cell therapy investigations. The research unveiled that the cultivated organoids bore a striking resemblance to human brain architecture, complete with neuroepithelial and matrix-like regions. At the molecular level, these organoids preserved hallmark mutations of the GALC gene, displayed diminished GALC enzyme activity, and accumulated psychosine, effectively recapitulating the defining features of Krabbe disease. Employing brain organoids as a model, Josse et al embarked on a comparative analysis of the transduction efficiencies of AAV5 and AAV9, uncovering that AAV5 outperformed AAV9 in this regard,^5^ a discovery that resonates with our observations. Expanding upon this, our comprehensive assessment of 11 distinct AAV serotypes in brain organoids pinpointed AAV5 as the frontrunner, with AAV6 and AAV2 trailing closely behind. Ultimately, our investigative efforts have endorsed AAV5 as the superior vector for brain organoid transduction, bolstering its status as a prime candidate for Krabbe disease gene therapy. The successful expression and restoration of GALC enzyme activity, as well as the reduction in psychosine levels after treatment with AAV5-CBA-hGALC, verify the potential of AAV5 as a feasible vector for therapeutic intervention.

In summary, we have pioneered a brain organoid model derived from iPSCs obtained from a patient with Krabbe disease. Through meticulous examination, we assessed the transduction efficiencies of 11 prevalent AAV serotypes within these organoids. Our findings revealed that AAV5 outshines its counterparts with the highest transduction efficiency. Building on this discovery, we adeptly harnessed AAV5 to deliver the *GALC* gene, catalyzing a significant resurgence in GALC enzyme activity and curtailing the buildup of psychosine, the toxic substrate associated with Krabbe disease, within the organoids (Fig. 1H). This groundbreaking study not only furnishes a robust human disease model that will propel Krabbe disease research forward but also introduces a promising viral vector, offering a potential gene therapy strategy for combatting this challenging disorder.

## Ethics declaration

The study was approved by the ethics committee of China–Japan Friendship Hospital (Title of the approved project: Therapeutic research on AAV-GALC for the treatment of iPS/neuron cells in patients with globoid cell leukodystrophy. Approval number: 2022-KY-035). All methods were carried out in accordance with relevant guidelines and regulations.

## Author contributions

Conceptualization: Y.L. and W.W.; methodology: Y.L., Z.C., and H.L.; investigation: Y.L., Z.C., J.W., H.L., M.W., Y.H., C.C., and X.L.; supervision: W.W.; writing original draft: Y.L.; writing-review & editing: Y. Lv and W.W.

## Funding

This work was funded by grants from the Beijing Municipal Science & Technology Commission (China) (No. Z211100002921005 to W.W.), National High-Level Hospital Clinical Research Funding (China) (No. 2022-NHLHCRF-PY-12 to WW), Hubei Provincial Department of Education's Scientific and Technological Research Project (China) (No. Q20211207 to Y.L.), Yichang Medical and Health Science and Technology Project (Hubei, China) (No. A22-2-069 to Y.L.), and the Open Foundation of Hubei Province Key Laboratory of Tumor Microenvironment and Immunotherapy (China) (No. 2023KZL08 to Y.L.).

## Conflict of interests

The authors declared that the research was conducted in the absence of any commercial or financial relationships that could be construed as a potential conflict of interest.

## References

[1] Wenger D.A., Rafi M.A., Luzi P. (2016). Krabbe disease: one hundred years from the bedside to the bench to the bedside. J Neurosci Res.

[2] Wenger D.A., Luzi P., Rafi M.A. (2021). Advances in the diagnosis and treatment of Krabbe disease. Int J Neonatal Screen..

[3] Wang W., Lv Y.F., Zhang Y.J., Dong W.J., Zhang Y. (2020). Generation of a human induced pluripotent stem cell line PUMCi001-A from a patient with Krabbe disease. Stem Cell Res.

[4] Mendell J.R., Al-Zaidy S.A., Rodino-Klapac L.R. (2021). Current clinical applications of *in vivo* gene therapy with AAVs. Mol Ther.

[5] Depla J.A., Sogorb-Gonzalez M., Mulder L.A. (2020). Cerebral organoids: a human model for AAV capsid selection and therapeutic transgene efficacy in the brain. Mol Ther Methods Clin Dev.

