# Supplementary material for: Identification of AAV serotypes for gene therapy in Krabbe iPSCs-derived brain organoids
Source: Genes Dis. 2024 Mar 19;12(1):101269. doi: 10.1016/j.gendis.2024.101269 (PMC11555342; doi:10.1016/j.gendis.2024.101269)
Supplement: Multimedia component 1 [file mmc1.docx]

**Fig.S1**

*GALC* mutations in the Krabbe brain organoids and H9 brain organoids. Genetic sequencing showed compound heterozygous mutations in the GALC gene (c.461 C > A, c.1244G > A) in Krabbe brain organoids.


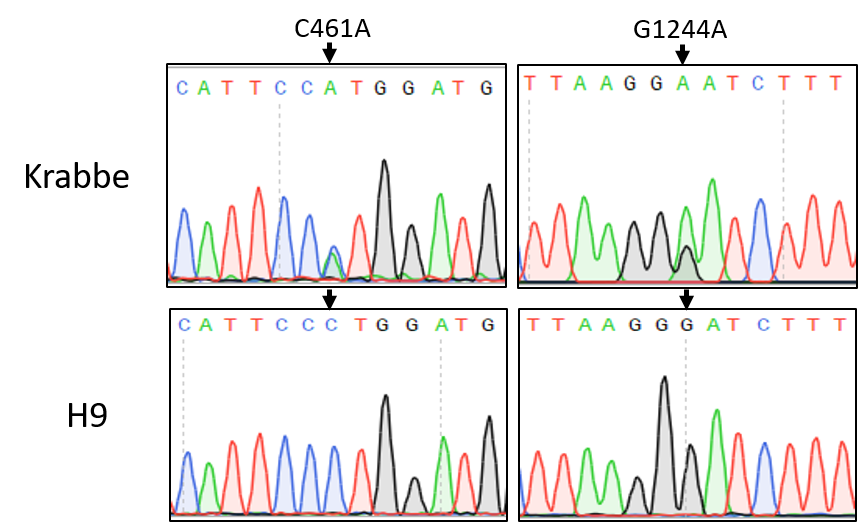


**Fig.S2**

GALC enzymatic activity in the H9 brain organoids and Krabbe brain organoids. H9: H9 human embryonic stem cell line, Data are expressed as the mean ± SD (n = 3). *** p < 0.001, ***p < 0.001.


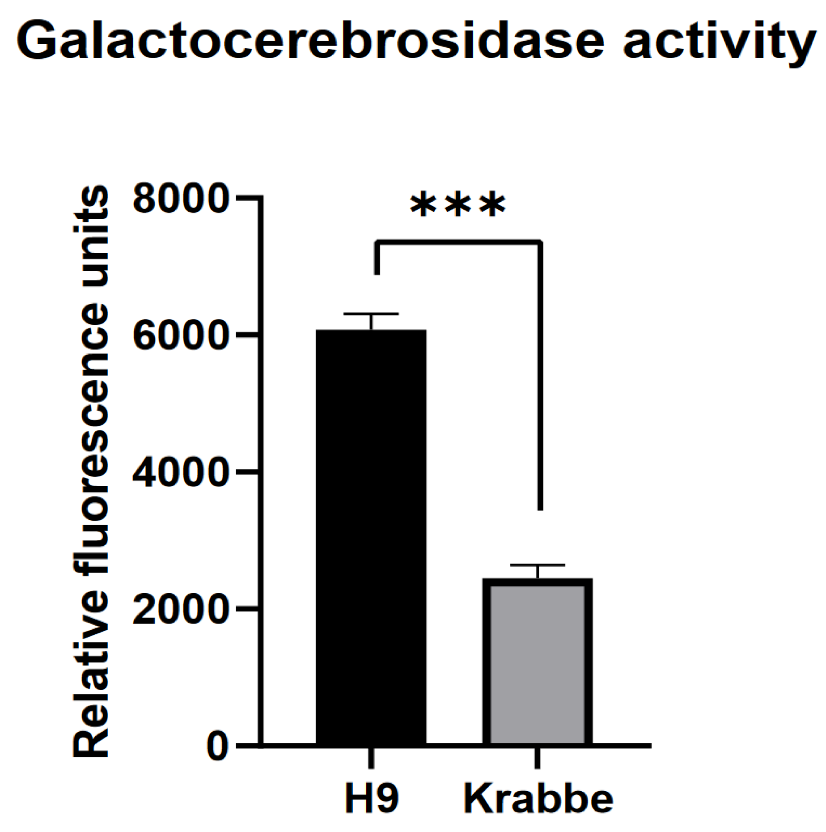


**Fig.S2**

Psychosine levels measured in the H9 brain organoids and Krabbe brain organoids. Data are expressed as the mean ± SD (n = 3). *** p < 0.001, ***p < 0.001.


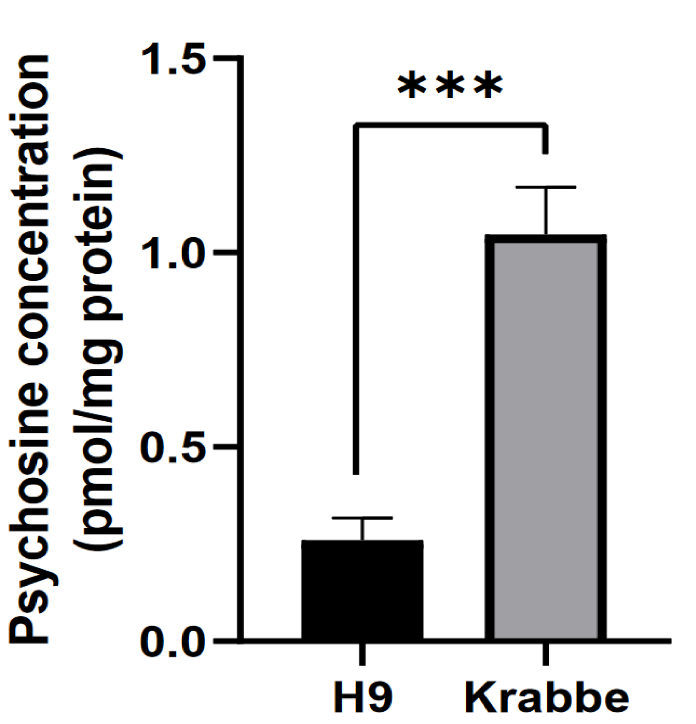


**METHODE**

**Generation of Brain Organoids Using Krabbe hiPSCs**

iPSCs were cultured on a matrix-coated culture plate with mTeSR1 medium (STEMCELL Technologies) at a temperature of 37°C and a CO_2_ concentration of 5%. Every 3-4 days, the cells were passaged using ReLeSR (STEMCELL Technologies). The brain organoids were generated according to the instructions of the STEMdiff Brain Organoid Kit (STEMCELL Technologies). iPSCs were dissociated into single-cell suspension using a cell dissociation reagent and seeded into ultra-low attachment 96-well plates with EB seeding medium. On the 2nd and 4th day after seeding, 100μl of EB formation medium was supplemented to each well. On the 5th day, the EBs were transferred to 24-well low attachment plates with Induction Medium and cultured for 3 days. Subsequently, the EBs were embedded in Matrigel and cultured in Expansion Medium in 6-well low attachment plates for 3 days. And then, the EBs were transferred to Maturation medium. Place plate of organoids on an orbital shaker in a 37°C incubator，Perform a full medium change every 3-4 days，By day 40, organoids will exhibit dense cores with regions of the organoid displaying optically translucent edges, and will typically be ready for analysis.

**Immunostaining of Brain Organoids**

The brain organoids of Krabbe patient was fixed with 4% PFA overnight at 4 °C. After dehydration, it was embedded in paraffin and sliced into 4μm sections. The tissue was then deparaffinized and rehydrated in a series of xylene and ethanol (100%, 95%, 70%). The sections were incubated in permeabilization buffer (0.1% v/v Triton X-100 in PBS) for 1.5 h at room temperature, followed by washing with PBST (0.1% v/v Tween20 in PBS) 3 times for 5 min, each. After blocking with 5% BSA at room temperature for 1 hour, it was incubated overnight at 4°C with the primary antibody diluted in blocking buffer. Blocking buffer was prepared by adding BSA (3% w/v) in permeabilization buffer. The antibodies and their dilutions used in this study are as follows: Tuj1 (Biolegend, MMS-435P, 1:200), Olig2 (Proteintech, 13999-1-AP, 1:200), Ki67 (Abcam, ab15580, 1:200), GFAP (Proteintech, 16825-1-AP, 1:200), SOX2 (chemicon, AB5603, 1:200), MAP2 (CST, 4542, 1:200), PAX6 (Biolegend, PRB-278P,1:300). After washing three times with PBST, the samples were incubated with fluorescently conjugated secondary antibodies (Alexa Fluor 488 and 594 conjugates, Invitrogen, 1:500) at room temperature for 1 hour. After washing three times with PBST again, the samples were mounted on glass slides. Fluorescence was detected and imaged using confocal microscopy (Nikon,A1R+).

**Selection of AAV Serotype for Brain Organoids**


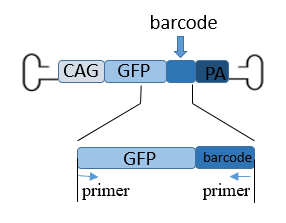
The AAV serotypes were screened according to the manufacturer's instructions using the AAV serotype rapid screening kit (Vigene Biosciences, SS000000). Briefly, microinjections were performed using needles with long continuous tapers. 2-3 positions were selected based on the size of the brain organoids and the number of buds. AAVmix was injected into the organoids, corresponding to 5E8-1E9 AAV genome copies (GC) of vector per brain organoid. After one week, RNA was extracted from the brain organoids, and reverse transcription was performed to convert RNA into cDNA. qPCR was then conducted using serotype-specific primers to determine the proportion of AAV in the brain organoid after viral infection. The AAVmix viral genome served as the control. A higher value indicates a higher infection efficiency of the respective serotype. The diagram below illustrates the AAV vector DNA elements, barcode position, and qPCR primer targeting sites.

**Generation of AAV5-hGALC Vector**

The human GALC cDNA was cloned into the pAAV-MCS vector, which was generously provided by Professor Ye Zhang from Peking Union Medical College. The recombinant genome consisted of AAV2 inverted terminal repeat sequences, along with the human cytomegalovirus enhancer/chicken β-actin promoter, human GALC cDNA, His tag, and the bovine growth hormone polyA signal, with a total length of approximately 3.6 kb. Subsequently, the entire expression vector was sequenced. To package the recombinant genome into AAV5 capsids, a triple-transfection procedure was performed using chimeric AAV2-Rep/AAV5-Cap and helper plasmids[1]. The virus was purified using iodixanol gradient centrifugation and dialyzed against PBS using a centrifugation column with a 100 kD cutoff (Millipore, MA, USA). Genomic titers of the recombinant AAV serotype were determined using quantitative PCR.

**Western Blot**

AAV5-hGALC vector was injected into brain organoids using the same injection method as for selection of AAV Serotype. The viral dose was 2E9 AAV genome copies (GC) of vector per brain organoid. The organoids were collected 48 hours after viral injection. Whole cell lysates were prepared using RIPA buffer (150 mM NaCl, 1% Triton X-100, 0.5% sodium deoxycholate, 0.1% SDS, 50 mM Tris, pH 8.0) supplemented with protease and phosphatase inhibitors. Western blot analysis was performed using the previously described method[2]. In brief, the samples were separated by 10% SDS-PAGE and then transferred onto nitrocellulose membranes. The membranes were blocked with 5% non-fat milk in Tris-buffered saline with 0.05% Tween (TBST) overnight with the primary antibody. The His-tagged galactosylceramidase was detected with a monoclonal anti-His antibody (1:5000; Proteintech). The membranes were washed three times with TBST and then incubated with horseradish peroxidase (HRP)-conjugated secondary antibody (Santa Cruz, 1:3000) for one hour. The proteins were further detected using ECL Western blot system.

**GALC activity assay**

GALC activity was assessed using the Lysosomal Galactocerebrosidase (GALC) Analysis Kit (catalog number ab253371, abcam), as previously described[3]. Briefly, brain organoids were lysed in lysis buffer A. The resulting lysates were centrifuged at 27,000×g for 30 min, and the supernatants were aspirated from the tubes. Next, 50 µg of total protein from each sample was transferred into different substrate tubes, and the total volume was adjusted to 100 µL with reaction buffer. After mixing the contents of the substrate tubes, the tubes were incubated at 37 °C for 2 h in the dark. The reactions were terminated immediately in stop buffer, and the terminated reaction mixtures were transferred to separate opaque 96-well plates. Fluorescence was detected using a fluorescence microplate reader, with an excitation wavelength of 365 nm and an emission wavelength of 454 nm.

**Psychosine Quantification**

Psychosine quantification was performed as previously described[4]. Fresh brain organoids were placed in a 10 mM sodium phosphate buffer solution (containing 0.1% NP40). After processing with a Bioprep-24 homogenizer and cell sonicator, psychosine was extracted from the tissue lysate (200 μg) using a methanol-acetic acid solution (0.5% acetic acid in methanol). Using D-erythro-Sphingosine (123-78-4, MCE) as an internal standard, the content of psychosine was determined using a triple quadrupole mass spectrometer coupled with a high-performance liquid chromatography system (1290 Infinity II+6470B, Agilent).

**Statistical analysis**

Data were analyzed with GraphPad Prism version 8. Differences between two groups were analyzed by the two-tailed Student’s t test, and differences between more than two groups were analyzed by ANOVA followed by Tukey’s test. Differences were considered significant for p values <0.05 (∗p < 0.05, ∗∗p < 0.01, and ∗∗∗p < 0.001). All error bars represent the SEM.

1. Pan, X., et al., *An Engineered Galactosylceramidase Construct Improves AAV Gene Therapy for Krabbe Disease in Twitcher Mice.* Hum Gene Ther, 2019. **30**(9): p. 1039-1051.

2. Tian, G., et al., *rAAV2-Mediated Restoration of GALC in Neural Stem Cells from Krabbe Patient-Derived iPSCs.* Pharmaceuticals (Basel), 2023. **16**(4).

3. Lv, Y., et al., *Identifying altered developmental pathways in human globoid cell leukodystrophy iPSCs-derived NSCs using transcriptome profiling.* BMC Genomics, 2023. **24**(1): p. 210.

4. Weinstock, N.I., et al., *Macrophages Expressing GALC Improve Peripheral Krabbe Disease by a Mechanism Independent of Cross-Correction.* Neuron, 2020. **107**(1): p. 65-81.e9.
